# Supplementary material for: Distinct vaginal microbial signatures in pregnancies complicated by antiphospholipid syndrome: depletion of Lactobacillus johnsonii and enrichment of Bifidobacterium dentium
Source: Microbiol Spectr. 2026 Mar 31;14(5):e03882-25. doi: 10.1128/spectrum.03882-25 (PMC13141996; doi:10.1128/spectrum.03882-25)
Supplement: Supplemental figure legends — Legends for Figures S1 to S4. [file spectrum.03882-25-s0001.docx]

**Figure S1. Comparison of vaginal microbiome profiles between Pre-APS and Index-APS**
(A) Principal coordinates analysis (PCoA) based on OTU-level Bray–Curtis distances showing no significant difference in overall community structure between Pre-APS and Index-APS samples. Ellipses indicate 95% confidence intervals. (B) Relative abundances of dominant bacterial species in Pre-APS and Index-APS groups (left) and differences between proportions with 95% confidence intervals (right); no taxa showed statistically significant differences (P values indicated). (C) Phylogenetic tree highlighting taxa associated with Index-APS.

**Figure S2. Rarefaction and pan–core OTU analyses**
(A)Rarefaction curves showing sampling depth for the Shannon index. (B)Pan OTU analyses showing number of total OTUs. (C)Core OTU analyses showing shared OTUs across APS and control groups.

**Figure S3. Venn Diagram in phyla, genera, species and OTUs**

(A-D) Venn diagrams showing the overlap of microbial taxa at the phylum, genus, species, and OTU levels between the APS and control groups.

**Figure S4. Association Between Risk Score and Clinical Characteristics**

(A-D) Scatter plots showing correlations between risk scores and Platelet count, APTT, Gravidity and gestational age at delivery, assessed using Spearman’s correlation.
