# Supplementary figures and images for: Distinct vaginal microbial signatures in pregnancies complicated by antiphospholipid syndrome: depletion of Lactobacillus johnsonii and enrichment of Bifidobacterium dentium
Source: Microbiol Spectr. 2026 Mar 31;14(5):e03882-25. doi: 10.1128/spectrum.03882-25 (PMC13141996; doi:10.1128/spectrum.03882-25)

A

PCoA on OTU level  
R=-0.02, P=0.633

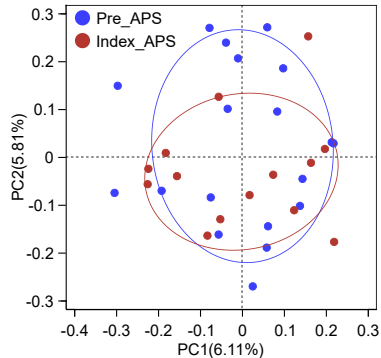

B

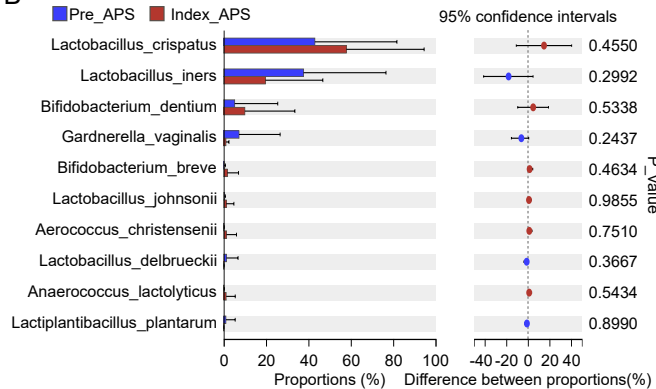

C

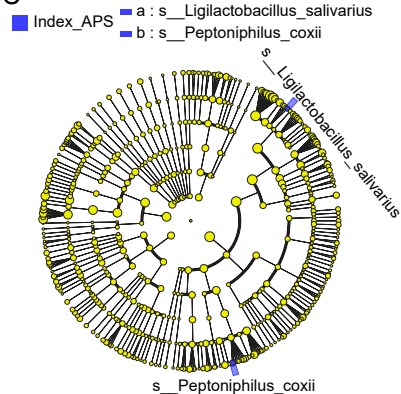

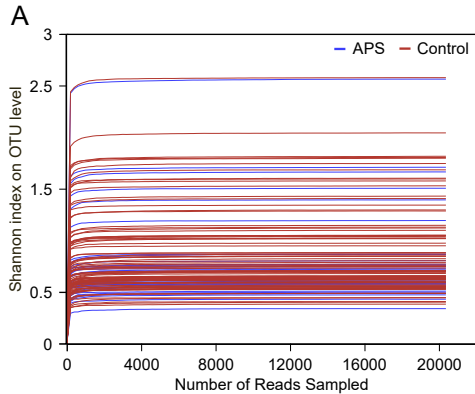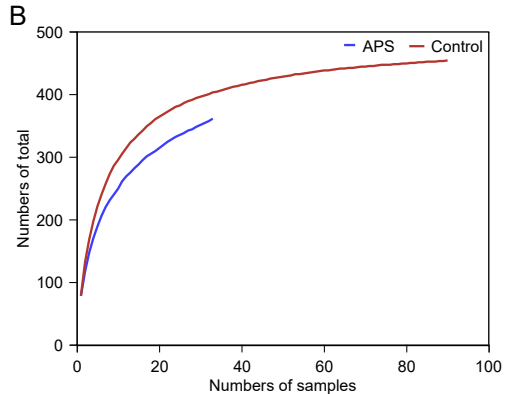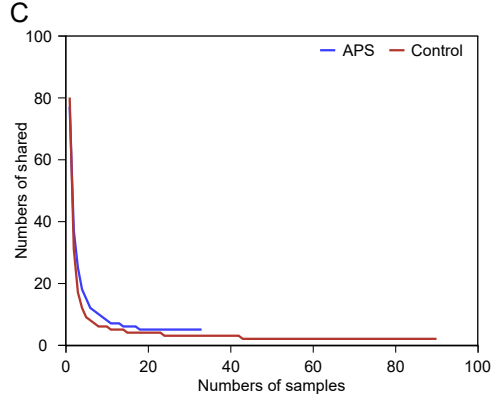

A

Phylum

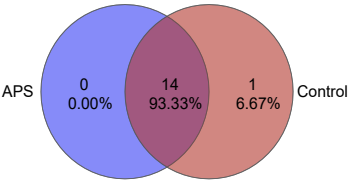

B

Genus

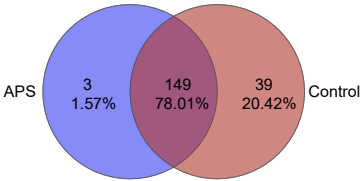

C

Species

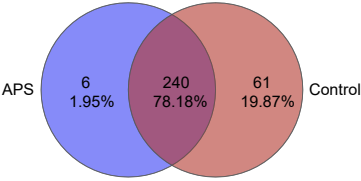

D

OTU

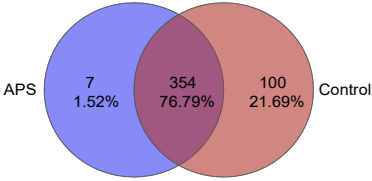

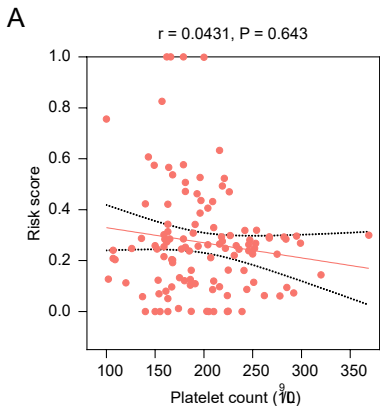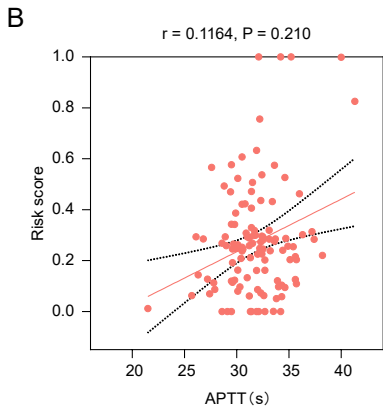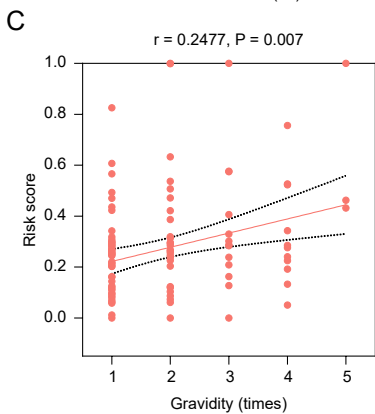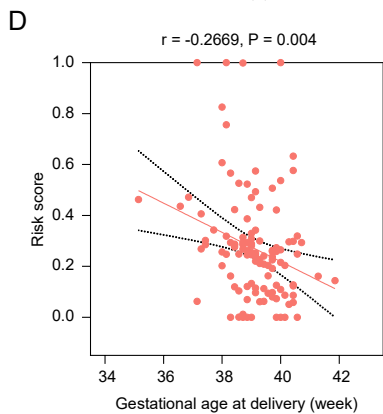

Supplement: Supplemental figures — Figures S1 to S4. [file spectrum.03882-25-s0002.pdf]
